# The gut microbiota instructs the hepatic endothelial cell transcriptome

**DOI:** 10.1016/j.isci.2021.103092

**Published:** 2021-09-10

**Authors:** Henning Formes, Joana P. Bernardes, Amrit Mann, Franziska Bayer, Giulia Pontarollo, Klytaimnistra Kiouptsi, Katrin Schäfer, Sebastian Attig, Teodora Nikolova, Thomas G. Hofmann, Jörn M. Schattenberg, Hristo Todorov, Susanne Gerber, Philip Rosenstiel, Tobias Bopp, Felix Sommer, Christoph Reinhardt

**Affiliations:** 1 Center for Thrombosis and Hemostasis (CTH), University Medical Center Mainz, Johannes Gutenberg-University Mainz, Langenbeckstrasse 1, 55131 Mainz, Germany; 2 Department of Chemistry, Biochemistry, Johannes Gutenberg-University Mainz, Hanns-Dieter-Hüsch-Weg 17, 55128 Mainz, Germany; 3 Institute of Clinical Molecular Biology, Christian-Albrechts-University and University Medical Center Schleswig-Holstein, Campus Kiel, 24105 Kiel, Germany; 4 Department of Cardiology, Cardiology I, University Medical Center Mainz, Johannes Gutenberg-University Mainz, Langenbeckstrasse 1, 55131 Mainz, Germany; 5 Research Center for Immunotherapy (FZI), University Medical Center, Johannes Gutenberg-University Mainz, Langenbeckstrasse 1, 55131 Mainz, Germany; 6 TRON, Translational Oncology at the University Medical Center, Johannes Gutenberg-University Mainz gGmbH, Freiligrathstrasse 12, 55131 Mainz, Germany; 7 Institute of Toxicology, University Medical Center Mainz, Johannes Gutenberg-University Mainz, 55131 Mainz, Germany; 8 Metabolic Liver Research Program, Department of Internal Medicine I, University Medical Center, Johannes Gutenberg University Mainz, Langenbeckstrasse 1, 55131 Mainz, Germany; 9 Institute of Human Genetics, University Medical Center, Johannes Gutenberg-University Mainz, Langenbeckstrasse 1, 55131 Mainz, Germany; 10 Institute for Immunology, University Medical Center Mainz, Johannes Gutenberg-University Mainz, Langenbeckstrasse 1, 55131 Mainz, Germany; 11 German Center for Cardiovascular Research (DZHK), Partner Site Rhine-Main, Mainz, Germany

**Keywords:** Hepatology, Microbiome, Transcriptomics

## Abstract

The gut microbiota affects remote organ functions but its impact on organotypic endothelial cell (EC) transcriptomes remains unexplored. The liver endothelium encounters microbiota-derived signals and metabolites via the portal circulation. To pinpoint how gut commensals affect the hepatic sinusoidal endothelium, a magnetic cell sorting protocol, combined with fluorescence-activated cell sorting, was used to isolate hepatic sinusoidal ECs from germ-free (GF) and conventionally raised (CONV-R) mice for transcriptome analysis by RNA sequencing. This resulted in a comprehensive map of microbiota-regulated hepatic EC-specific transcriptome profiles. Gene Ontology analysis revealed that several functional processes in the hepatic endothelium were affected. The absence of microbiota influenced the expression of genes involved in cholesterol flux and angiogenesis. Specifically, genes functioning in hepatic endothelial sphingosine metabolism and the sphingosine-1-phosphate pathway showed drastically increased expression in the GF state. Our analyses reveal a prominent role for the microbiota in shaping the transcriptional landscape of the hepatic endothelium.

## Highlights

• Germ-free mice show transcriptome differences in the liver sinusoidal endothelium
• Gut microbiota suppresses sphingolipid metabolism in the hepatic sinusoidal endothelium
• Cholesterol flux and angiogenesis in liver endothelium is microbiota-regulated
• *Bacteroides thetaiotaomicron* did not affect expression levels of the identified genes

## Graphical abstract

**Figure:**
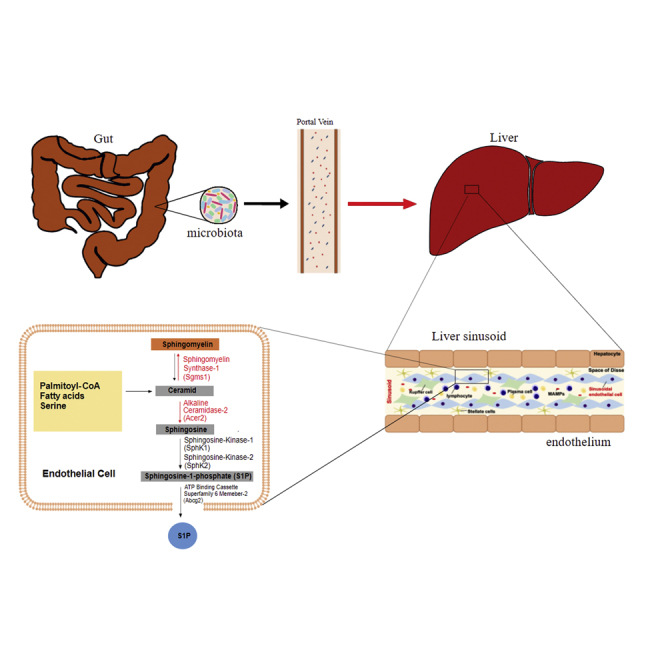

## Introduction

Commencing at birth, all animals are stably associated with symbiotic microbial communities (microbiota) and are to be regarded as metaorganisms (Esser et al., 2019). Therefore, the host and its microbiota are no longer viewed as separate entities but they interact through multiple pathways and thus coevolve (Koh and Bäckhed, 2020). For example, the microbiota provides enzymatic functions that yield essential metabolites used by the host (Sonnenburg et al., 2005; Baxter et al., 2019). Vice versa, the microbiota also shapes the expression profile of metabolic enzymes of the host and thus modulates nutrient harvest and use (Poole et al., 2019; Sommer et al., 2015). This complex interplay is strongly determined by nutritional intake and contributes to a variety of metabolic disease phenotypes (Schroeder and Bäckhed, 2016; Kübeck et al., 2016).

The endothelia of different organs are highly specialized (Augustin and Koh, 2017). However, so far, the involvement of the microbiota in the specification of organotypic endothelial cell (EC) functions remains unexplored. The gut microbiota shapes intestinal vascular physiology locally, by inducing adaptive remodeling of intricate capillary networks in small intestinal villus structures (Reinhardt et al., 2012). Interestingly, the microbiota also impacts remote vascular phenotypes, such as the vascular function of large conductance vessels or the formation of cerebral cavernous malformations in cerebrovascular disease (Karbach et al., 2016; Tang et al., 2017). Microbiota-derived signaling compounds and metabolites readily enter the liver compartment through the portal circulation, and thereby influence the hepatic and systemic inflammatory tone (Balmer et al., 2014; Clarke et al., 2010; Schaupp et al., 2020). The liver is steadily exposed to gut microbial products via its specialized microvasculature and thus represents a predilection site for the regulation of host metabolism (Busch et al., 2017; Johnson et al., 2020).

The collective metabolic capacities of the gut microbiota and the gastrointestinal tract are linked via the hepatic portal circulation with the specialized sinusoidal vascular bed of the liver (Ascher et al., 2020). Thus, the liver is unique in its involvement in nutrient uptake and key host metabolic functions, ensuring immunovigilance, e.g. by residing Kupffer cells or the release of acute phase proteins (Hickey and Kubes, 2009; Jäckel et al., 2017). Of note, sinusoidal ECs are the most abundant non-parenchymal cell type in the liver. The sinusoidal endothelium, containing fenestrae forming a mechanical sieve with gaps of approximately 100 nm in diameter, together with the hepatocytes (parenchymal epithelium), form a specialized niche compartment, the perisinusoidal space (space of Disse), harboring mesodermal stellate cells (Asahina et al., 2009; Sawitza et al., 2009). Hepatic sinusoids are not only a primary site to combat invading microorganisms (Massberg et al., 2010) but also provide a specialized endothelium that under steady-state conditions can influence the extent of arterial thrombus growth, dependent on the microbiota (Jäckel et al., 2017; Kiouptsi et al., 2019). Interestingly, the ECs of hepatic capillaries lack a basement membrane, have an extremely high endocytotic capacity, serve important roles in antigen presentation and leukocyte recruitment, and form permeable fenestrations that enable the exchange of solutes and the uptake of lipoproteins (Shetty et al., 2018; Schurich et al., 2009; Fraser et al., 2012). Since the hepatic endothelium steadily encounters microbiota-derived patterns and metabolites that strongly affect its metabolic capacity and the recruitment of immune cells (Faraj et al., 2019; Balmer et al., 2014; Busch et al., 2017), our goal was to pinpoint the microbiota-regulated gene fraction of the hepatic endothelial transcriptome. Until now, the microbiota’s impact on the transcriptomic landscape of the hepatic microvascular endothelium, a central element of the gut-liver axis (Tripathi et al., 2018), is poorly understood.

## Results

### The liver sinusoidal EC transcriptome of germ-free mice is distinct from conventionally raised mice

**Figure 1:**
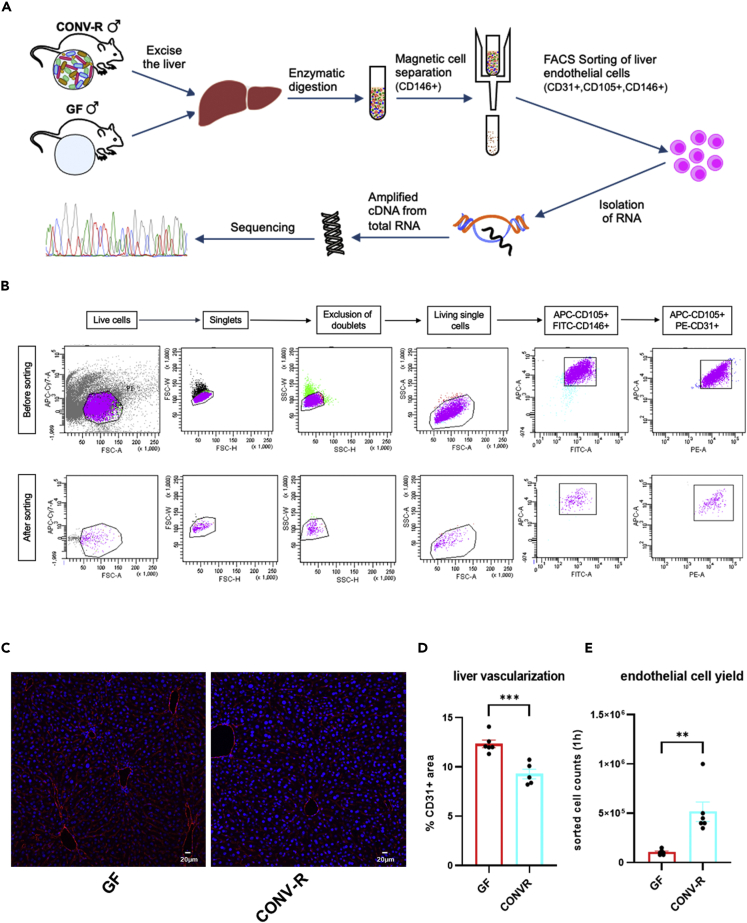
Purification protocol of hepatic sinusoidal endothelial cells and immunofluorescence analysis of the vascularization of germ-free (GF) vs. conventionally raised (CONV-R) mouse livers (A) Overview of the experimental set up and the different endothelial markers for EC isolation. The liver of male GF and CONV-R mice was excised, followed by an enzymatic digestion to yield single-cell suspensions. Magnetic cell sorting (CD146^+^) was followed by FACS sorting (CD31^+^,CD105^+^,CD146^+^), isolation of RNA, amplification of cDNA from total RNA and finally the RNA-sequencing. (B) FACS sorting strategy for the purification of endothelial cells from GF and CONV-R mouse livers. The gating strategy started from live cells, inclusion of singlets and exclusion of doublets. From these living single cells, positive endothelial cells were selected with a double control. (C) Immunofluorescence analysis of endothelial cells in livers of GF and CONV-R mice. A representative image in a magnification of 200× is shown (scale bar: 20 μm). Staining with the endothelial marker CD31^+^ is red. Cell nuclei are stained with TO-PRO-3 iodide (blue). (D) Calculation of the liver vascularization from GF and CONV-R mice of CD31^+^ area using cellSens Dimension Desktop software. Results are presented as mean ± S.E.M. (E) Analysis of the sorted liver endothelial cell yields from GF and CONV-R mice after 1 h. Results are presented as mean ± S.E.M. ∗∗ = p < 0.01; ∗∗∗ = p < 0.005.

To address this pivotal question, we dissociated the livers of age-matched male germ-free (GF) and conventionally raised (CONV-R) specific pathogen-free (SPF) C57BL/6J mice into single-cell suspensions and purified hepatic ECs via magnetic cell sorting (MACS) for the EC marker CD146 (melanoma cell adhesion molecule, MCAM), followed by fluorescence-activated cell sorting (FACS) to select for CD31^+^ (platelet-EC adhesion molecule-1, PECAM-1), CD105^+^ (endoglin), and CD146^+^ ECs (Abel et al., 2013) (Figure 1A). While CD31 is a pan-endothelial marker for ECs and CD105 is a marker for the hepatic sinusoidal ECs, known to display a very low expression of selectins (Steinhoff et al., 1993; El Filali et al., 2013; Abel et al., 2013), we chose CD146 both for coated magnetic beads and FACS as it specifically binds to hepatic microvascular ECs. Applying this sorting strategy, we gained highly pure EC preparations (98%) with a yield of approximately 2 × 10^6^ cells after MACS and 6 × 10^4^ to 1 × 10^6^ cells after FACS per mouse liver (Figure 1B). Interestingly, livers of GF mice showed an increased vascularized area as compared with CONV-R counterparts, demonstrated by quantitative analyses of immunofluorescence staining for the EC marker PECAM-1 (CD31) (Figures 1C and 1D). However, the isolation protocol yielded approximately 5-times lower numbers of purified FACS sorted ECs obtained from GF mouse livers as compared with CONV-R controls (Figure 1E). From these purified ECs, we extracted total RNA of an average amount of 466 ng (CONV-R) and 30 ng (GF). Concentration and quality of the RNAs were assessed by Qubit analysis and whole-transcriptome amplification (WTA) was carried out using the Ovation RNA-Seq System V2 kit with 20 ng total RNA as input for first-strand cDNA synthesis (see STAR Methods). Next, RNA-sequencing was performed on an Illumina HiSeq 4000 sequencer using TruSeq DNA Nano Kit, which yielded an average of 9 million single-end reads (1 × 50 bp). Overall, we were able to detect 1537 differentially expressed genes (DEG, p.adjust <0.05 combined with Log2FC) in ECs of GF versus CONV-R mice. 932 of these DEGs displayed an increased expression in the GF compared with CONV-R mice, whereas 605 DEGs had decreased expression.

### Transcripts of the sphingolipid metabolism are enriched in the liver sinusoidal endothelium of GF mice

**Figure 2:**
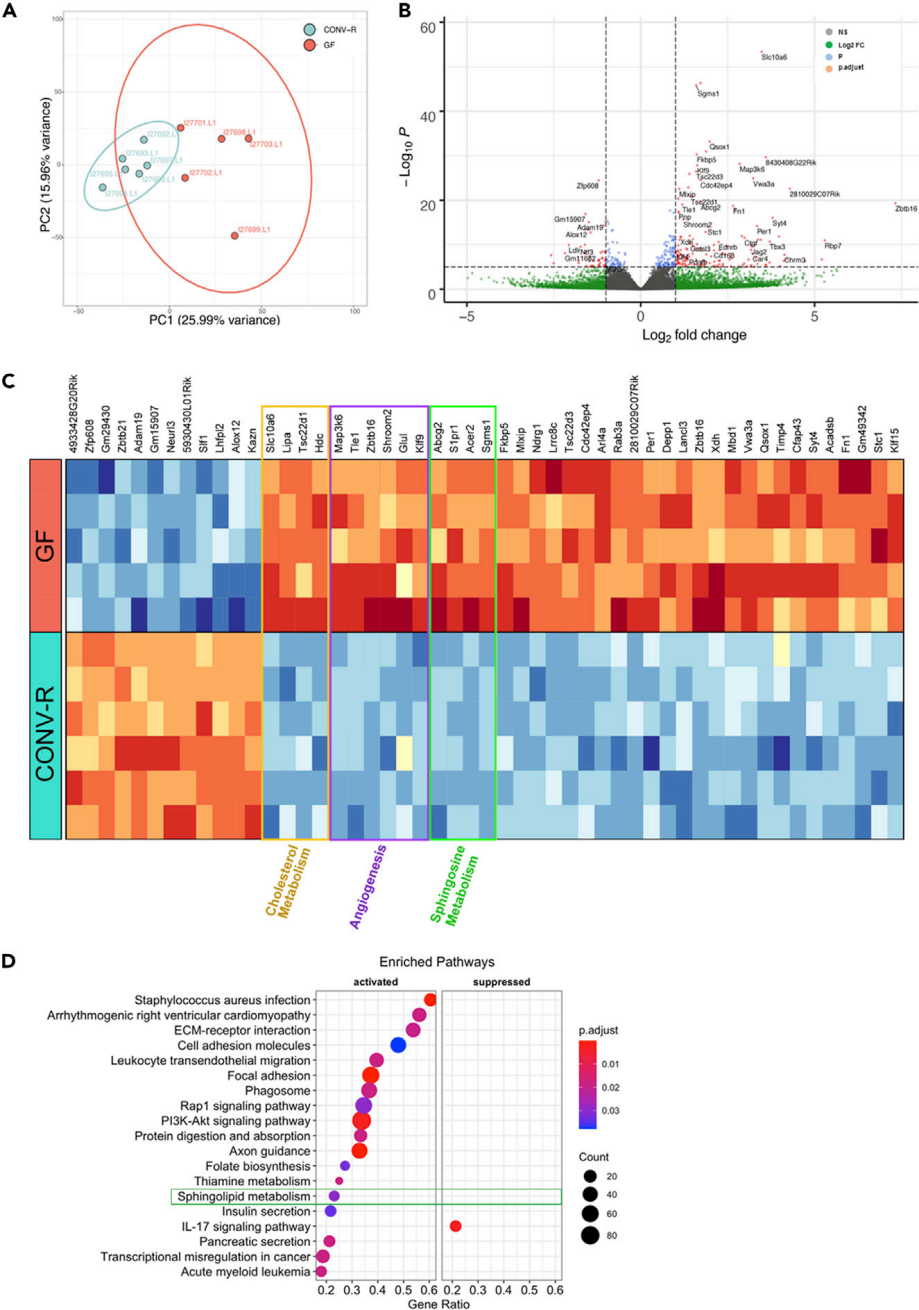
Comparative analysis of RNA-sequencing data of isolated liver sinusoidal endothelial cells of germ-free (GF) vs. conventionally raised (CONV-R) C57BL/6J mice (A) Principle component analysis (PCA) of the sequenced GF (red, n = 5) vs CONV-R (turquoise, n = 6) mice. PC1 shows a variance of 25.99% and PC2 of 15.96%. (B) Volcano Plot of differentially expressed genes (DEGs). The genes are color coded according to difference in expression (Log2 fold change, Log2FC) and significance (adjusted p value, p.adjust). Gray: no difference in expression; not significant. Green: difference in expression, but not significant. Blue: significant difference, but the expression change is within [−1,1] Red: significant difference and high range expression change. (C) Heatmap of the top 50 differentially expressed genes in hepatic endothelium comparing GF with CONV-R mice. The gold, violet and green frames highlight DEGs involved in cholesterol metabolism, angiogenesis and sphingosine metabolism, respectively. (D) Gene set enrichment analysis of KEGG pathways. 19 KEGG pathways were significantly enriched at GF housing conditions. Pathways with a positive enrichment score were considered to be activated. The size of the dots corresponds to the number of genes in the reference gene set. The gene ratio is equal to the number of genes in the leading-edge subset divided by the total size of the gene set. The color of the dots corresponds to the adjusted p value (p.adjust; Benjamini-Hochberg method). See also Figures S1, S2, S4, and Table S1.

Next, we aimed to identify those transcripts in the microvascular hepatic endothelium that were most significantly up- or downregulated by the presence or absence of a microbiota. Principal component analysis identified the GF and CONV-R samples as separate clusters (Figure 2A). As illustrated in the volcano plot (Figure 2B), in hepatic sinusoidal ECs of male GF mice, expression of the solute carrier family 10 member 6 (*Slc10A6*) transporter was most strongly upregulated (p.adjust = 6.4 × 10^−50^, Log2FC = 3.47). SLC10A6 transports taurolithocholic acid-3-sulfate and sulfoconjugated pyrenes. Transcript levels of the sphingomyelin-synthase 1 (*Sgms1*) gene were also significantly increased in GF hepatic sinusoidal ECs (p.adjust = 7.9 × 10^−43^, Log2FC = 1.59). *Sgms1* encodes for the enzyme transferring the phosphatidyl-headgroup of phosphatidylcholine on the primary hydroxyl of ceramide to yield the sphingolipid sphingomyelin. Other strongly upregulated transcripts were those of quiescin sulfhydryl oxidase 1 (*Qsox1*) (p.adjust = 2.3 × 10^−30^, Log2FC = 1.98), catalyzing the oxidation of sulfhydryl groups to disulfides with reduction of oxygen to hydrogen peroxide and FKBP prolyl isomerase 5 (*Fkbp5*) (p.adjust = 1.2 × 10^−27^, Log2FC = 1.61), which is involved in protein folding and trafficking. In contrast, in the hepatic sinusoidal endothelium from CONV-R mice there was a significant upregulation of the transcription factor that suppresses NNF609 transcription (*Zfp608*) (p.adjust = 4.1 × 10^−22^, Log2FC = 1.21), the disintegrin and metalloproteinase domain-containing 19 (*Adam19*) that is involved in cell migration and cell-matrix interaction (p.adjust = 3.7 × 10^−13^, Log2FC = 1.49), the neutralized E3 ubiquitin protein ligase 3 (*Neurl3*) (p.adjust = 3.1 × 10^−13^, Log2FC = 1.00), and of arachidonate 12-lipoxygenase (*Alox12*) (p.adjust = 1.4 × 10^−11^, Log2FC = 1.50), an enzyme involved in the generation of eicosanoids and lipoxins. Collectively, these data highlight the functional role of the microbiota in the transcriptional regulation of hepatic microvascular endothelium.

All identified liver endothelial transcripts that were more than 2-fold increased comparing GF with CONV-R mice and reached a significance level of p.adjust <0.05 (Figure 2B) were presented in a heatmap (Figure 2C) and assigned to their respective functions with regard to EC physiology (Table S1). Thus, by analyzing gene card entries (Rebhan et al., 1998) for the identified up- and downregulated transcripts, and by the analysis of published functions of those genes with regard to EC physiology, we revealed biochemical pathways influenced by the microbiota.

Interestingly, we identified transcripts engaged in transcriptional regulation (*Meis2*, *Zfp608*, *Zbtb21* upregulated in CONV-R versus Klf1 upregulated in GF), angiogenesis (*Map3k6*, *Ccn2*, *Tle1*, *Zbtb16*, *Cdkn1a*, *Shroom2*, *Dusp1*, *Glul*, and *Klf9*, all upregulated in GF), circadian rhythmicity (*Klf15* and *Per1* upregulated in GF), and cell adhesion (*Kank2*, *Adam19*, and *Kazn* upregulated in CONV-R versus *Fn1*, and *Timp4* upregulated in GF) (Figure 2C).

Although the liver cholesterol-lowering function of the gut microbiota has long been recognized (Wostmann and Wiech, 1961), mainly depending on facilitated excretion caused by microbial biohydrogenation to hydrophilic coprostanol (Pontarollo et al., 2020), the microbiota’s impact on the cellular regulation of hepatic endothelial cholesterol flux remains unresolved. We identified several genes, which determine cholesterol flux, as upregulated in liver ECs of GF mice relative to their CONV-R counterparts (Figure 2C). In contrast to a previous study showing the regulation of the Na^+^-dependent bile acid transporter solute carrier family 10 member 6 (*Slc10a6*) via lipopolysaccharide signaling, depending on the nuclear receptor farnesoid X receptor (FXR) and the retinoid X receptor (Kosters et al., 2016; Sawkat Anwer and Stieger, 2014), we found increased expression of *Slc10a6* at GF housing conditions. In line with increased plasma lipoprotein levels at chow diet at GF housing conditions (Kiouptsi et al., 2019), expression of *Tsc22d1*, encoding a transcription factor that is critically involved in the formation of high-density lipoprotein (HDL) particles (Jäger et al., 2014), was increased in the hepatic endothelium of GF mice. Moreover, *lipase A*, coding for the enzyme hydrolyzing cholesteryl esters and triglycerides in the lysosome, that is a known regulator of the mTOR pathway and the endothelial barrier (Zhao et al., 2014, 2017), was upregulated at GF housing conditions.

Strikingly, our transcriptome analysis revealed a highly significant microbiome-dependent suppression of gene functions that mediate the endogenous synthesis of sphingosine-1-phosphate (S1P). The S1P synthesis pathway in the liver sinusoidal endothelium of GF mice was consistently upregulated (Figures 2B and 2C; Table S1). Key enzymes of the sphingolipid metabolism such as sphingomyelin-synthase-1 (*Sgms1*) that catalyzes the conversion of sphingomyelin to ceramide, alkaline ceramidase-2 (*Acer2*), converting ceramide to the signaling active metabolite S1P, the S1P-transporter ATP-binding cassette superfamily 6 member 2 (*Abcg2*), and also the 7-helix G-protein coupled S1P-receptor (*S1pr1*) were upregulated at GF housing conditions, demonstrating that this pathway is effectively suppressed by the presence of microbiota (Figures 2B and 2C; Table S1). The S1P pathway is a pivotal regulatory pathway of vascular function. S1P signaling is critically involved in endothelial proliferation and cell migration (Kimura et al., 2000), angiogenesis (Cyster and Schwalb, 2012), cell survival (Kwon et al., 2001), acts pro-inflammatory and ensures control of the endothelial barrier (McVerry and Garcia, 2004; Feistritzer and Riewald, 2005).

In support of the microbiota-dependent regulation of several pathways in the sinusoidal liver endothelium, gene set enrichment analysis (GSEA) revealed 19 Kyoto Encyclopedia of Genes and Genomes (KEGG) pathways that were significantly enriched. All pathways, except for the IL-17 signaling pathway, were activated in GF relative to CONV-R mice (Figure 2D). The distribution of Log2FC of the genes associated with each pathway is shown in Figure S1A. Strikingly, GSEA identified a highly significant microbiome-dependent suppression of gene functions that mediate the endogenous synthesis of S1P. The sphingolipid metabolism pathway in the sinusoidal liver endothelium of GF mice was significantly upregulated (Figures 2D and S1B). The running enrichment score and the ranked list metric for the sphingolipid metabolism pathway are shown in Figure S1B.

**Figure 3:**
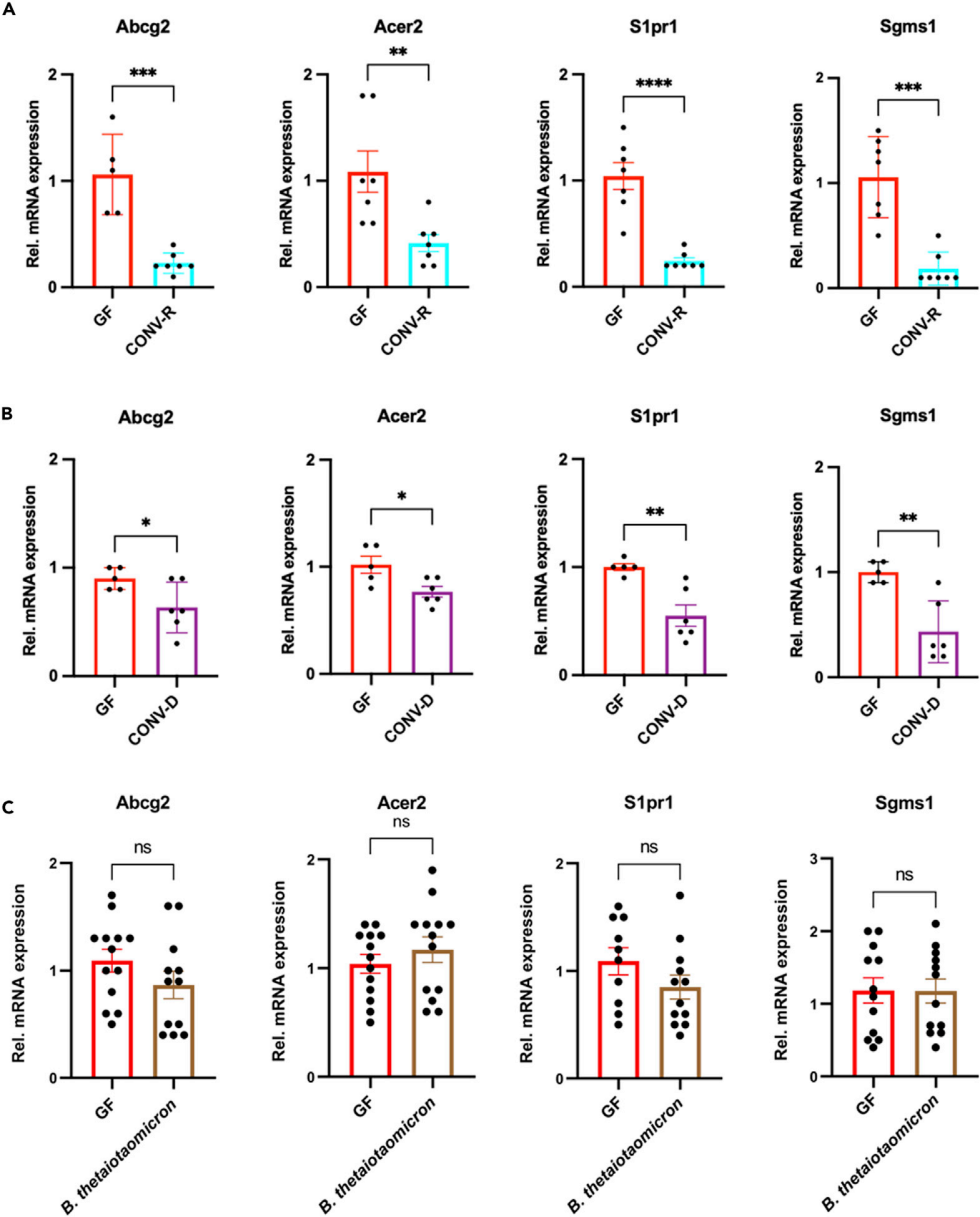
Relative mRNA expression of identified microbiota-regulated genes of the sphingolipid metabolism in MACS-isolated hepatic endothelial cells at various colonization conditions (A) qRT-PCR quantification of mRNA levels of *Abcg2*, *Acer2*, *S1pr1*, and *Sgms1* in MACS-sorted liver endothelial cells of germ-free (GF; red bars; N = 5–7 mice per group) vs. conventionally raised mice (CONV-R, turquoise bars; N = 7 mice per group); male mice were analyzed. (B) qRT-PCR quantification of mRNA levels of *Abcg2*, *Acer2*, *S1pr1*, and *Sgms1* in MACS-sorted liver endothelial cells comparing germ-free (GF; red bars; N = 5 mice per group) vs. conventionally derived mice (CONV-D, purple bars; N = 6 mice per group); 4 weeks colonization period, mixed groups were analyzed. (C) qRT-PCR quantification of mRNA levels of *Abcg2*, *Acer2*, *S1pr1*, and *Sgms1* in MACS-sorted liver endothelial cells comparing germ-free (GF; red bars; N = 10–13 mice per group) vs. *Bacteroides thetaiotaomicron*-monocolonized mice (brown bars; N = 12–13 mice per group); 4 weeks colonization period, male mice were analyzed. See also Figure S3. Results are presented as mean ± S.E.M. ∗ = p < 0.05; ∗∗ = p < 0.01; ∗∗∗ = p < 0.005; ∗∗∗∗ = p < 0.001.

**Figure 4:**
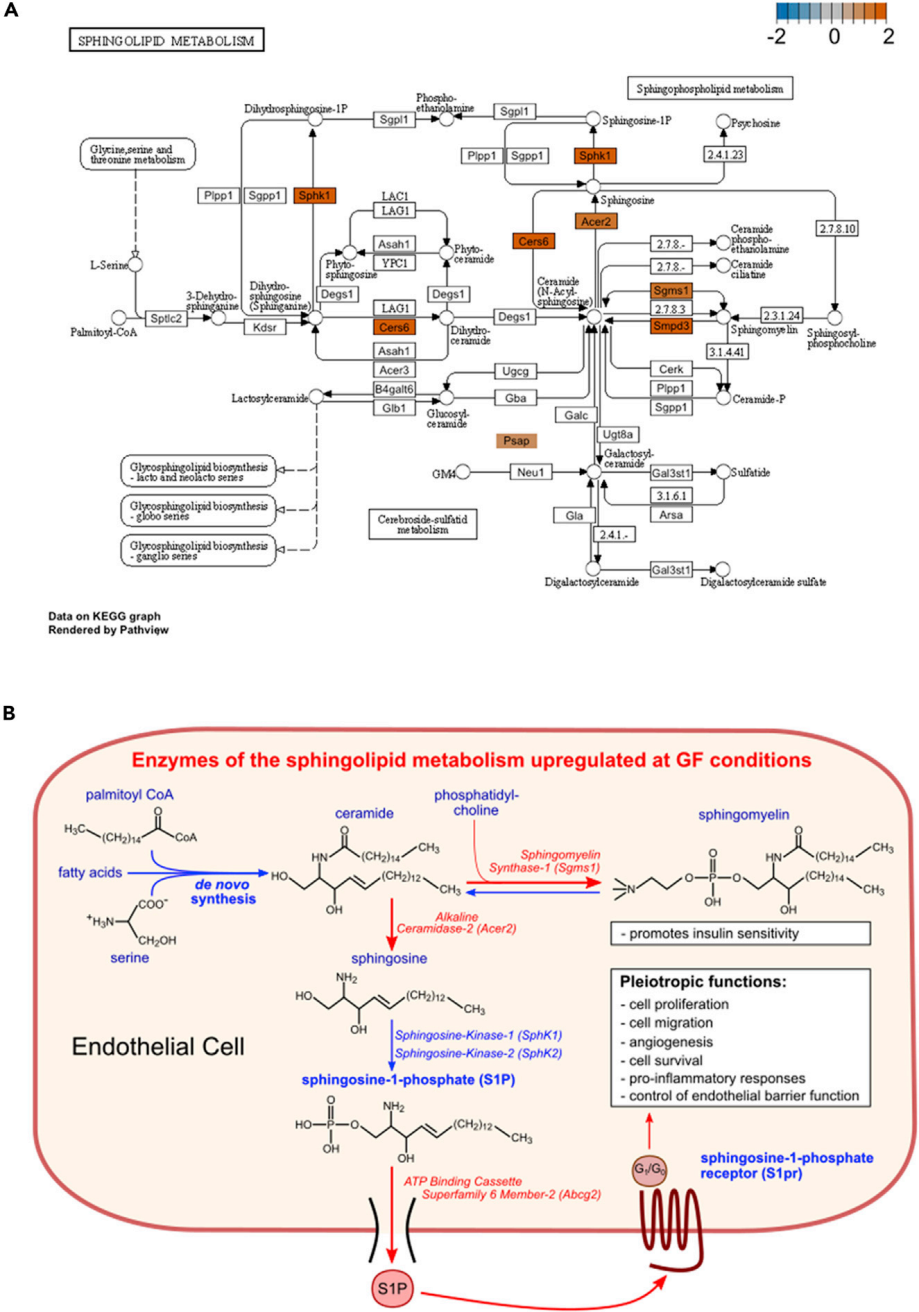
Pathway analysis identifies an upregulation of sphingolipid metabolism in hepatic sinusoidal endothelium at germ-free housing conditions (A) Sphingolipid metabolism Kyoto Encyclopedia of Genes and Genomes (KEGG) pathway visualization. Differentially expressed genes of the sphingolipid KEGG pathway are colored according to their Log2 fold change (Log2FC). Positive Log2FC values (red) indicate genes that are upregulated in germ-free (GF) mice relative to conventionally raised (CONV-R) mice. (B) Illustration of the sphingolipid metabolism found upregulated in hepatic endothelial cells of GF mice relative to CONV-R counterparts.

Using the online tool REVIGO, we performed separate gene ontology (GO) enrichment analyses for the 932 upregulated and 605 downregulated DEGs (Supek et al., 2011). Thus, we categorized the resulting GO terms (p.adjust <0.01) according to their biological processes, cellular components and molecular functions. Redundant GO terms were removed and the remaining terms were grouped by relevant categories. In the “Biological Process” category (Figure S2, blue bars), transcripts upregulated in GF mice (absence of bacteria) were significantly enriched for functions related to the regulation of phosphate metabolism, regulation of protein modification, regulation of cell proliferation, enzyme linked receptor protein signaling, reactive oxygen species biosynthetic process, regulation of signal transduction, innate immune response, immune system process, tube morphogenesis, and apoptotic process. In contrast, CONV-R housing conditions led to an increased expression of genes identified with GO terms for cellular response to stress, cell division, vesicle docking, regulation of adherence junction organization, DNA conformation change, pyrimidine deoxyribonucleoside triphosphate, pentose-phosphate shunt (non-oxidative branch), positive regulation of alcohol biosynthesis, establishment of protein localization, and smoothened signaling pathway. In addition to the analysis of “Biological Process”, we performed a GO term analysis for “Cellular Component” (Figure 1, Figure 2, Figure 3, Figure 4orange bars) and “Molecular Function” (Figure S2, gray bars). In summary, the identified differentially expressed genes, involving all three GO term categories, suggest that the functionality of the microvascular liver endothelium is strongly affected by the host colonization status.

### Conventionalization experiments confirm microbiota-induced suppression of the hepatic endothelial sphingolipid synthesis pathway

In line with our RNA-sequencing data, qRT-PCR analyses on independent MACS-sorted liver sinusoidal EC isolations confirmed reduced mRNA expression of *Abcg2*, *Acer2*, *S1pr1*, and *Sgms1* in CONV-R mice relative to their GF counterparts (Figure 3A). Conventionalization of GF mice by a 4-week colonization with the cecal content harvested from a CONV-R mouse (conventional-derived; CONV-D) confirmed that the suppression of the sphingolipid synthesis pathway is a dynamic and colonization-induced process (Figure 3B). Similar to CONV-R mice, CONV-D mice showed reduced expression levels of *Abcg2*, *Acer2*, *S1pr1*, and *Sgms1* relative to GF controls. Next, we monocolonized GF mice with the gram-negative anaerobe *Bacteroides thetaiotaomicron*, a common symbiont of the lower intestine (Xu et al., 2003), to check if even colonization with a single microbial species yielded similar results. Notably, *B. thetaiotaomicron* was chosen for monocolonization, as this bacterium induces angiogenesis and vascular remodeling in the small intestine (Stappenbeck et al., 2002). Monocolonization of GF C57BL/6J mice was confirmed by PCR-amplification of a 721 bp product with *B. thetaiotaomicron*-specific 16S rDNA primers (Figure S3A and S3B) (Teng et al., 2000). However, monocolonization of GF mice was insufficient to suppress these components of the hepatic endothelial sphingolipid metabolism (Figure 3C).

In support of our RNA-sequencing data (Figure 2C), qRT-PCR analyses demonstrated a microbiota-dependent reduction of the mRNA expression of *Slc10A6*, *Lipa*, and *Tsc22d1* in isolated liver sinusoidal ECs, both in the GF vs CONV-R comparison and in the conventionalization experiment (Figures S4A and S4B). Similar to the microbiota’s impact on sphingolipid metabolism, the regulation of genes involved in cholesterol flux was not dependent on *B. thetaiotaomicron*, as monocolonization with this gut bacterium did not alter the mRNA expression levels of the identified microbiota-regulated genes (Figure S4C). Thus, our results identified the colonization-dependent suppression of distinct elements of the hepatic endothelial sphingolipid pathway and of genes involved in cholesterol flux.

### KEGG pathway analysis highlights the upregulation of key elements of hepatic endothelial sphingolipid metabolism

Visualization of the Kyoto Encyclopedia of Genes and Genomes (KEGG) sphingolipid metabolism pathway emphasized the important regulatory role of the gut microbiota on key enzymes of the hepatic endothelial sphingolipid pathway. Importantly, *Sgms1* and *Smpd3*, both of which catalyze the conversion of sphingomyelin to ceramide, were upregulated at GF housing conditions (Figure 4A). Furthermore, *Acer2*, which converts ceramide to sphingosine and *Sphk1*, catalyzing the phosphorylation of sphingosine to the signaling active metabolite S1P, was also overexpressed in the liver sinusoidal ECs of GF mice (Figures 2B and 4A), demonstrating that the presence of microbiota effectively suppresses this pathway.

Taken together, our analyses indicate that GF housing conditions favor the conversion of ceramide to sphingomyelin by upregulation of *Sgms1,* as well as the conversion of ceramide to sphingosine by *Acer2* (Figure 4B). Furthermore, our results suggest that in the liver sinusoidal endothelium of GF mice, the release of S1P and endothelial S1P-receptor expression might be enhanced.

## Discussion

Our study on GF and colonized C57BL/6J mice defined the liver sinusoidal endothelium as a vascular bed that is subjected to intense microbiome-induced transcriptome changes, affecting the expression of genes involved in metabolism, angiogenesis, circadian rhythmicity, signaling processes and sphingolipid metabolism. Based on colonization experiments, our study put focus on the microbiota’s role in regulating the sphingolipid synthesis pathway in the hepatic sinusoidal endothelium, identifying a new metabolic aspect of microbiota-host interaction in the vascular gut-liver axis.

The here uncovered upregulation of the sphingolipid synthesis-pathway in the sinusoidal liver endothelium of GF mice (Figures 2C, 3A, 3B, and 4) is intriguing as these data complement a recent metabolomics study (Johnson et al., 2020). Importantly, the identified enzymes of the sphingolipid metabolism were confirmed by qRT-PCR in independent EC isolations (Figures 3A and 3B). Both, functional scoring analysis (GSEA), as well as pathway assignment based on gene card entries and KEGG pinpointed the regulation of hepatic endothelial sphingolipid synthesis by the gut microbiota (Figures 2D and 4A; Table S1). Our results add to the seminal work of Johnson et al. (Johnson et al., 2020), who recently identified Bacteroidetes (i.e. *B. thetaiotaomicron*), the major phylum of the mammalian gut microbiota, as a relevant source of sphingolipids. This recent study demonstrated that, dependent on microbial palmitoyltransferase functions, microbiota-derived sphingolipids are transferred to the epithelial lining of the intestine and taken up via the portal vein, impacting host ceramide and sphingomyelin levels in the liver. Our results imply that in the absence of microbiota, the host’s capacity of endogenous sphingosine synthesis is enhanced by the liver endothelium to ensure S1P-signaling, which is in line with the suppression of the endogenous synthesis pathway by exogenous *Bacteroides*-derived sphingolipids, i.e. by gut bacterial sphinganine (Johnson et al., 2020). Thus, our transcriptomics data are in agreement with the results from a gut epithelial Caco-2 cell culture model and in line with gnotobiotic monocolonization experiments testing the impact of *Bacteroides thetaiotaomicron* wild-type and mutant strains on GF Swiss Webster mice, confirming the microbiota’s regulatory role as a rich source of sphingolipids. Microbiota-derived sphingolipids can exert immunological properties, as described for the glycosphingolipid α-galactosylceramide produced by *Bacteroides fragilis*, an agonist of CD1d-restricted natural killer T cells (Wieland Brown et al., 2013). However, in contrast to conventionalization with a cecal gut microbiota from a CONV-R donor mouse, our monocolonization experiments with *B. thetaiotaomicron* on GF C57BL/6J mice did not suppress the identified microbiota-regulated transcripts *Abcg2*, *Acer2*, *S1pr1*, and *Sgms1* as part of the sphingolipid synthesis pathway. This suggests that other gut resident bacteria may regulate the expression of enzymes of the hepatic endothelial sphingolipid pathway.

In conclusion, our comparative whole-transcriptome analysis on GF vs. CONV-R mice unambiguously demonstrates that hepatic ECs are a prominent site for the microbiota’s action on host sphingosine-metabolism but also on other key metabolic processes (e.g. cholesterol flux, bile acid metabolism), and angiogenesis (Figures 2C, 2D, and 3; Table S1). Considering the broad influence of the gut microbiota on sphingosine-metabolism, both as a signaling-relevant source of sphingolipids (Wieland Brown et al., 2013; Johnson et al., 2020) and through the regulation of the expression of key functions of that meta-organismal lipid metabolic pathway, it is of paramount importance to delineate how this microbiota-host interaction integrates into the physiology of different vascular beds. The revealed link between the microbiota and hepatic endothelial function is of high translational value as the S1P pathway affects cardiovascular disease development and vascular thrombosis (Mahajan-Thakur et al., 2015).

Evidence linking the intestinal microbiome to the pathophysiology of liver disease is steadily increasing. Most importantly, dysbiosis in non-alcoholic fatty liver disease (NAFLD), constituting the most prominent liver pathology, has been linked to disease stage and treatment response (Mouzaki and Loomba, 2020). In patients with NAFLD, diet and exercise have profound effects on the gut microbiome (Armandi and Schattenberg, 2021; Huber et al., 2019). Gut microbiota signatures have been explored as biomarkers of disease severity and as markers in pharmacotherapy – in particular when involving bile acid metabolism (Loomba et al., 2021). The novel link of the microbiome and liver ECs, that we explored in the present study, expands the established role of liver ECs in regulating the innate immune system (Knolle and Wohlleber, 2016) and provides the rationale for future explorations. In perspective, the exploration of our findings on the microbiota-endothelial cross talk and in particular the hepatic endothelium in liver cirrhosis, portal hypertension and NAFLD seems warranted. Furthermore, since aging is characterized by the defenestration of the hepatic sinusoidal endothelium, a potential driver of disrupted lipid metabolism and atherosclerosis (Le Couteur et al., 2002), the functional genomics of this organotypic EC type awaits further exploration by analyzing aged gnotobiotic mouse models of hyperlipidemia.

### Limitations of the study

While a 98% purity was achieved by MACS sorting for the EC marker CD146 followed by FACS to select for CD31^+^, CD105^+^, and CD146^+^ ECs, the viability of the sorted cells was low. Therefore, to enable efficient sequencing, a WTA step to yield sufficient cDNA amounts was required. Although the microbiota-regulated transcripts were validated by qRT-PCR on independent samples, our study lacks comprehensive validation on the protein level. In future work, it will be most interesting to test the functional importance of endothelial sphingolipid synthesis and cholesterol flux by quantitative lipidomics analyses.

## STAR★ Methods

### Key resources table

**Table:** 

REAGENT or RESOURCE	SOURCE	IDENTIFIER
**Antibodies**		

Rabbit anti-mouse CD31 (PECAM-1)	Cell signaling Technology	Cat#: 77699
AF555 conjugated donkey anti-rabbit	Life Technologies	Cat#: A31572
FITC Anti-mouse CD146	Biolegend	Cat#: 134705 RRID:AB_2143526
PE anti-mouse CD31	Biolegend	Cat#: 102407 RRID:AB_312902
APC anti-mouse CD105	Biolegend	Cat#120413 RRID:AB_2277915
Fixable Viability Dye eFluor 780	Invitrogen	Cat#: 65-0865

**Bacterial and virus strains**		

*Bacteroides thetaiotaomicron*	DSMZ-German Collection of Microorganisms and cell cultures GmbH	Cat#: DSM2079

**Chemicals, peptides, and recombinant proteins**		

Dulbecco’s Modified Eagle Medium (DMEM) with glutamine	Thermo Fisher Scientific	Cat#: 61965-026
CD146 MicroBeads	Miltenyi Biotec	Cat#: 130-092-007
LS Column	Miltenyi Biotec	Cat#: 130-042-401
ReliaPrep™ RNA Tissue Miniprep System kit	Promega	Cat#: Z6112
High Sensitivity RNA ScreenTape System	Agilent	Cat#: 5067- 5579
Roti Histofix	Carl Roth	Cat#: P087
Dako Faramount Aqueous Mounting Medioum	Dako	Cat#: S3025
TO-PRO™-3 Iodide	Thermo Fisher Scientific	Cat#: T3605
iTaq Universal SYBR Green Supermix	Biorad	Cat#: 1725121
blocking solution	Dako	Cat#: S3022

**Critical commercial assays**		

Liver Dissociation Kit	Miltenyi Biotec	Cat#: 130-105-807
Quant-iT RNA HS Assay kit	Thermo Fisher Scientific	Cat#: Q32852
Ovation RNA-Seq System V2 kit	NuGen	Cat#: 7102
TruSeq DNA Nano Kit	Illumina	Cat#: 20015964
High Capacity cDNA Reverse Transcriptase Kit	Thermo Fischer Scientific	Cat#: 4368814

**Deposited data**		

RNA-seq data	This paper	Gene Expression Omnibus: GSE180520

**Experimental models: Organisms/strains**		

C57BL/6J	The Jackson Laboratory	Cat#: JAX:000664 RRID:IMSR_JAX:000664

**Oligonucleotides**		

Primers for qPCR see Table in Method details	Primer bank Harvard https://pga.mgh.harvard.edu/primerbank/	Thermo Fisher Scientific

**Software and algorithms**		

Trim Galore version 0.4.4		
STAR version 2.5.2b		
featureCounts version 1.5.2		
Graphpad version 9.1.2		
REVIGO	Java web application	http://revigo.irb.hr
clusterProfiler v3.16.1	Bioconductor	https://bioconductor.org/
pathview v1.28.1	Bioconductor	https://bioconductor.org/
Olympus cellSens Dimension Desktop	Olympus	https://www.olympus-lifescience.com/en/software/cellsens/

**R package Algorithms**		

DESeq2, stats version 3.6.3		
ggplot2 version 3.3.3		
EnhancedVolcano version 1.6.0		
gplots version 3.1.1		

**Other**		

gentleMACS C-Tube	Miltenyi Biotec	Cat#: 130-093-237
gentleMACS Octo Dissociator	Miltenyi Biotec	Cat#: 130-096-427

### Resource availability

#### Lead contact

Further information and requests for resources and reagents should be directed to and will be fulfilled by the lead contact, Christoph Reinhardt (Christoph.Reinhardt @unimedizin-mainz.de).

#### Materials availability

This study did not generate new unique reagents.

### Experimental model and subject details

#### Microbe strains

*Bacteroides thetaiotaomicron* (Cat#DSM2079) glycerol stock solutions were kept at -80°C. Before the gavage, an aliquot was cultured in Brain Heart Infusion Broth (Sigma Aldrich, Missouri, USA) at 37°C under continuous shaking (250 rpm) for 48 hours, until the optical density of the suspension at 600 nm (OD_600 nm_) reached 0.6.

#### Animals

C57BL/6J (RRID: IMSR_JAX:000664) mice were maintained as germ-free (GF) mouse colonies in sterile flexible film mouse isolator systems. The GF status of mice was verified every second week by 16S rDNA PCR and by culture testing. Conventionally raised (CONV-R) specific-pathogen-free (SPF) C57BL/6J mice originated from the same colonies. All the compared groups were fed the same standard laboratory diet. All experimental animals were 10 to 12 weeks old male mice housed in the Translational Animal Research Center (TARC) of the University Medical Center Mainz under SPF or GF conditions in EU (European Union) type II cages with 2 to 5 cage companions with standard autoclaved lab diet and water ad libitum, 22±2°C room temperature and a 12 hours light/dark cycle. All groups of mice were age-matched and free of clinical symptoms. For mono-association experiment, GF mice were colonized for 4 weeks with the bacterium *Bacteroides thetaiotaomicron* by oral gavage once with 200 μl of *B. thetaiotaomicron* culture. After a colonization period of 4 weeks, mice were sacrificed, and the liver was excised. GF C57BL/6J wild type animals were used as controls. For the conventionalization experiment (conventional-derived; CONV-D) cecal content of age-matched conventionally raised mice was suspended in 400 μl of sterile PBS. GF mice were gavaged with 100 μl of the suspension, and GF wild type C57BL/6J mice were used as controls. All procedures performed on mice were approved by the local committee on legislation on protection of animals (Landesuntersuchungsamt Rheinland-Pfalz, Koblenz, Germany; G17-1-075).

### Method details

#### Isolation of liver endothelial cells

Mice were sacrificed by cervical dislocation and the liver was excised. Liver endothelial cells were isolated using a Liver Dissociation Kit (Miltenyi BiotecBergisch Gladbach, Germany). The excised liver was transferred into a gentleMACS C-Tube (Miltenyi Biotec), the dissociation mix was added and the tubes were placed on the gentleMACS Octo Dissociator with Heaters for 34 minutes (Miltenyi Biotec). After termination of the program, the cell suspension ran through a MACS SmartStrainer (100 μm, Miltenyi Biotec) and was washed with 5 ml Dulbecco’s Modified Eagle Medium (DMEM) with glutamine (Thermo Fisher Scientific, Waltham, USA). The filter was discarded and the cell suspension was centrifuged at 300xg for 10 minutes. The supernatant was discarded and cells were resuspended in PEB buffer (phosphate-buffered saline (PBS), pH 7.2, 2 mM EDTA and 0.5% bovine serum albumin (BSA)).

#### Magnetic cell separation

The number of pelleted cells was determined with a Neubauer counting chamber and CD146 MicroBeads (Miltenyi Biotec) were added to the cell pellet volume in a 1:9 ratio. The cell suspension was mixed and incubated for 15 minutes in the refrigerator at 4°C. After the incubation, the cells were washed with 1 ml of PEB buffer and centrifuged at 300xg for 5 minutes. The supernatant was discarded. The LS Column (Miltenyi Biotec) was placed into the magnetic field of a MACS Separator and the column was prepared by rinsing with 3 ml of PEB buffer. The washed cells were resuspended in 500 μl of PEB Buffer and applied onto the LS Column. The protocol was followed by three washing steps with 3 ml of PEB Buffer each. After the washing step, the LS Column was removed from the MACS separator and placed on a suitable collection tube. 5 ml of PEB buffer were pipetted onto the column and all CD146^+^ cells were flushed out by using a plunger.

#### Flow cytometric analyses

Liver endothelial cells were identified using fluorophore-coupled antibodies. Cells were stained for the cell surface markers CD146 (Biolegend, San Diego, USA), CD31 (Biolegend, San Diego, California, USA), and CD105 (Biolegend) to identify liver sinusoidal endothelial cells (Strauss et al., 2017). To separate dead from living cells, we used the fixable viability dye eFluor 780 (eBioscience, San Diego, California, USA). Flow cytometric analysis and sorting of cells was performed with a BD FACSAria^TM^ II analytical flow cytometer (BD Biosciences, Franklin Lakes, New Jersey, USA).

#### Isolation of total RNA

For the isolation of total RNA we used the ReliaPrep™ RNA Tissue Miniprep System kit (Promega, Madison, Wisconsin, USA). RNA-Isolation was performed according to the manufacturer’s instructions.

#### Whole-transcriptome amplification and RNA sequencing

Concentration and quality of the isolated RNAs were assessed using the Quant-iT RNA HS Assay kit (Molecular Probes, Eugene, Oregon, USA) on a Qubit fluorometer (Thermo Fisher Scientific, Waltham, Massachusetts, USA) and using the High Sensitivity RNA ScreenTape System (Agilent, Santa Clara, California, USA) on a Tapestation (Agilent). Whole-transcriptome amplification (WTA) was carried out using the Ovation RNA-Seq System V2 kit (NuGen, Redwood City, California, USA) according to the manufacturer’s instructions with 20 ng total RNA as input for first-strand cDNA synthesis. The double-stranded cDNAs from the WTA were then used as input for the TruSeq DNA Nano Kit (Illumina, San Diego, California, USA) to prepare barcoded sequencing libraries following the manufacturer’s instructions with the exception of omitting the cDNA synthesis steps and directly proceeding to adapter ligation. All samples were sequenced using an Illumina HiSeq 4000 sequencer (Illumina) with an average of 9 million single-end reads (1x 50 bp) at IKMB NGS core facility (Institute of Clinical Molecular Biology, Christian-Albrechts-University, Kiel, Germany).

#### Gene ontology term analysis

For the calculation of semantic similarity measures between different GO terms, we used the tool REVIGO a server-side Java web application on a Glassfish 3 server. REVIGO is available from http://revigo.irb.hr. We used a small (0.5) similarity for our analysis and the database for the species *Mus musculus*. For the semantic similarity we used SimRel.

#### Gene set enrichment analysis

Gene set enrichment analysis (GSEA) of KEGG pathways was performed with clusterProfiler v3.16.1. The input genes were ranked according to their log fold change from the differential expression analysis. Genes with low counts as identified by the independent filtering step in the DESeq2 analysis were not included in the GSEA. Pathways with an adjusted p-value less than 0.05 were considered significantly enriched. Multiple comparisons adjustment was performed with the Benjamini-Hochberg method. Visualization of selected enriched KEGG pathways was facilitated with pathview v1.28.1.

#### Immunofluorescence staining

Mice were sacrificed and liver tissue was excised and fixed in 4% paraformaldehyde (Carl Roth, Karlsruhe, Germany) and processed for paraffin embedding at University Medical Center Mainz, Core Facility Histology. Sections were dewaxed and heat-induced epitope-retrieval was carried out using 10 mM citrate buffer, pH 6. Following blocking with ready to use blocking solution (Dako, Jena, Germany) for 30 minutes at room temperature, rabbit anti-mouse CD31 (PECAM-1) (Cell Signaling Technology, Danvers, Massachussets, USA) was applied at 1:100 (v/v) dilution in Dako blocking solution and incubated at 4^o^C overnight in a humid chamber. After washing the slides with PBS containing 0.1% (v/v) tween-20 (PBST), sections were incubated with AF555 conjugated donkey anti-rabbit secondary antibody (Life Technologies, Carlsbad, California, USA) at dilution 1:500 in blocking solution for 1 hour at room temperature. After washing, sections were counter stained with TO-PRO™-3 Iodide (ThermoFisher Scientific) at 1:100 (v/v) dilution in PBS for 30 minutes at room temperature. Slides were washed and mounted using Dako Faramount Aqueous Mounting Medium (Dako). Images were captured using a Zeiss Observer Z1 Axio LSM and ZEN 2009 imaging software. Vascularized area in comparision to the total area was determined using Olympus cellSens Dimension Desktop software.

#### Quantitative real-time polymerase chain reaction

Isolated mRNA from isolated liver endothelial cells of GF and CONV-R were converted into cDNA with High Capacity cDNA Reverse Transcriptase Kit (Life Technologies, Thermo Fisher Scientific, Waltham, USA). In brief, 2 μg of total RNA was mixed with 2 μl RT Buffer, 0.8 μL dNTP Mix, 2 μl RT Random Primers, 1 μl reverse transcriptase in a final volume of 20 μl. cDNA Transcription was performed according to the manufacturer’s instructions. The cDNA was diluted by adding 380 μl of RNase-free water. Relative mRNA Expression was quantified by qTOWER^3^ G (Analytic Jena AG, Jena, Germany) using iTaq Universal SYBR Green Supermix (Biorad Laboratories Inc., Hercules, California, USA). Reaction was performerd in the final volume of 25 μl according to the manufacturer’s instructions. Each analysis was performed in triplicates. Cycle threshold (Ct) values were analyzed. For calculation of relative mRNA expression levels, the mean values of the Ct values that were normalized against the house keeping gene GAPDH, were used. The primer sequences used are:

**Table:** 

Primer	Gene	Sequence [5′ → 3′]
Slc10A6 for	Solute Carrier Family 10 Member 6	GGAGGGCCATGCGAATCTAAA
Slc10A6 rev		TGTCAGAGGCATAAGTCCAAAC
Lipa for	Lysosomal acid Lipase A	TGTTCGTTTTCACCATTGGGA
Lipa rev		CGCATGATTATCTCGGTCACA
Tsc22d1 for	Transforming growth factor beta stimulated Protein TSC-22	CCAGTGGCGATGGATCTAGGA
Tsc22d1 rev		CTTGCACCAGAGCTATTGTCA
Abcg2 for	ATP-binding cassette subfamily G Member 2	CCAGTGGCGATGGATCTAGGA
Abcg2 rev		CTTGCACCAGAGCTATTGTCA
S1pr1 for	Sphingosine-1-phosphate receptor 1	ATGGTGTCCACTAGCATCCC
S1pr1 rev		CGATGTTCAACTTGCCTGTGTAG
Acer2 for	Alkaline Ceramidase 2	TGTGGCATATTCTCATCTGCCT
Acer2 rev		CAATAAAAGCCCATTTCTCGCTG
Sgms1 for	Sphingomyelin Synthase 1	TTGGCACGCTGTACCTGTATC
Sgms1 rev		CAGTCTCCAAAGAGCTTCGGA
GAPDH for	Glycerinaldehyd-3-phosphat-Dehydrogenase	TGCCACTCAGAAGACTGTGG
GAPDH rev		TTCAGCTCTGGGATGACCTT

### Quantification and statistical analysis

#### Statistical analysis

The RNA-seq data were processed using an in-house pipeline (https://github.com/nf-core/rnaseq). Briefly, adapters and low-quality bases from the RNA-seq reads were removed using Trim Galore (version 0.4.4). The filtered reads were mapped to the mouse genome (GRCm38) using STAR aligner (version 2.5.2b). One sample of the GF genotype was removed from further analyses due to a significantly lower number of mapped reads. Expression counts of the transcripts were estimated using featureCounts (version 1.5.2) and then normalized across samples using the DESeq (Love et al., 2014) normalization method. DEseq2 was also used to determine differentially expressed genes. Genes were considered significantly differentially expressed if the adjusted *p-value* (Benjamini-Hochberg multiple test correction method) was less than 0.05. The principal component analysis (PCA) plot was calculated using stats package (v 3.6.3) and the figure using ggplot2 (v 3.3.3). Volcano plots were used to visualize the differentially expressed genes highlighting expression changes between experimental groups, which was accompanied by a heat map based on individual sample Z-scores. The Volcano plot was designed using EnhancedVolcano (v 1.6.0), heatmap was made using gplots (v 3.1.1). The qPCR data were presented as mean values + standard error of the mean (SEM). For the evaluation of the difference between two groups Student’s unpaired t-test were used. Two groups were statistically compared with an ANOVA test to analyze the variance of the values. The p-values were considered statistically significant in case of p<0.05. Statistical calculation was performed with GraphPad Prism version 9.1.2 (GraphPad Software Inc, San Diego, California, USA). The sample size and statistical details of each experiment can be found on the figure legends.

## Data Availability

• RNA-seq data have been deposited at Gene Expression Omnibus (https://ncbi.nlm.nih.gov/geo). The accession number is GSE180520. • This paper does not report original code. • Any additional information required to reanalyze the data reported in this paper is available from the lead contact upon request.
