# Supplementary material for: The gut microbiota instructs the hepatic endothelial cell transcriptome
Source: iScience. 2021 Sep 10;24(10):103092. doi: 10.1016/j.isci.2021.103092 (PMC8479694; doi:10.1016/j.isci.2021.103092)
Supplement: Supplementary file 1 — Document S1. Figures S1–S4 and Table S1 [file mmc1.pdf]

iScience, Volume *24*

Supplemental information

The gut microbiota instructs the hepatic endothelial cell transcriptome

Henning Formes, Joana P. Bernardes, Amrit Mann, Franziska Bayer, Giulia Pontarollo, Klytaimnistra Kiouptsi, Katrin Schäfer, Sebastian Attig, Teodora Nikolova, Thomas G. Hofmann, Jörn M. Schattenberg, Hristo Todorov, Susanne Gerber, Philip Rosenstiel, Tobias Bopp, Felix Sommer, and Christoph Reinhardt

Supplemental Information

Figure S1

A

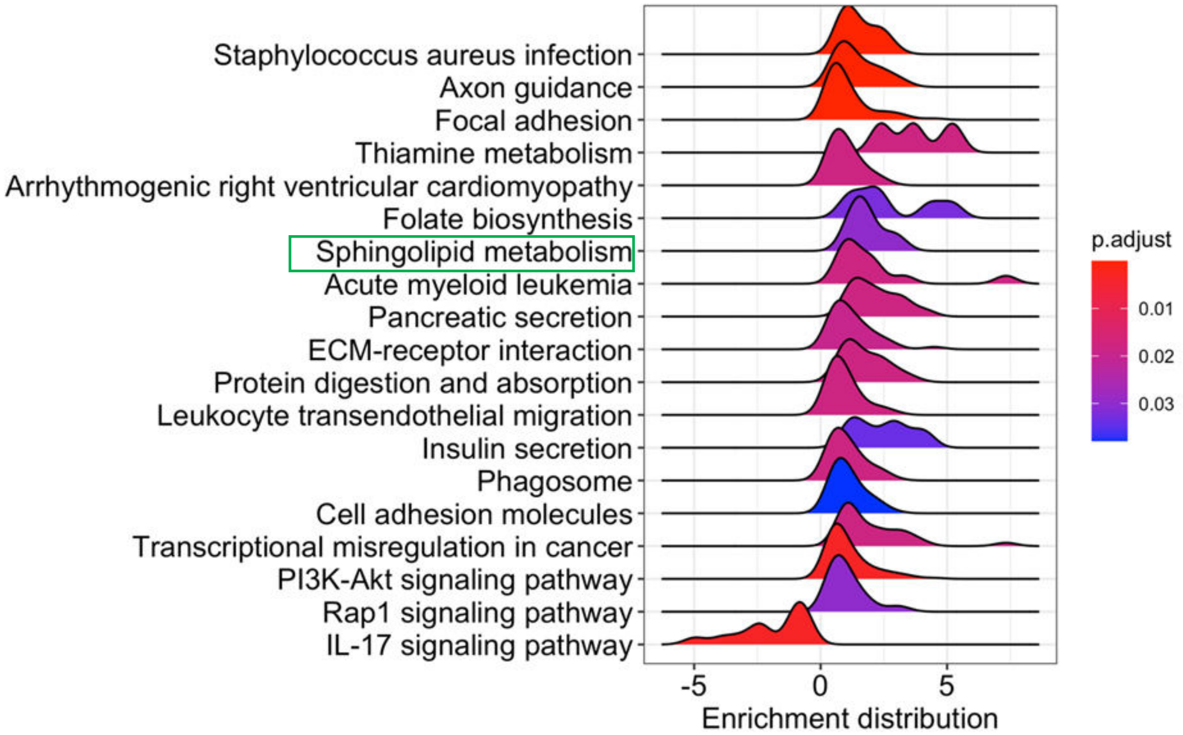

B

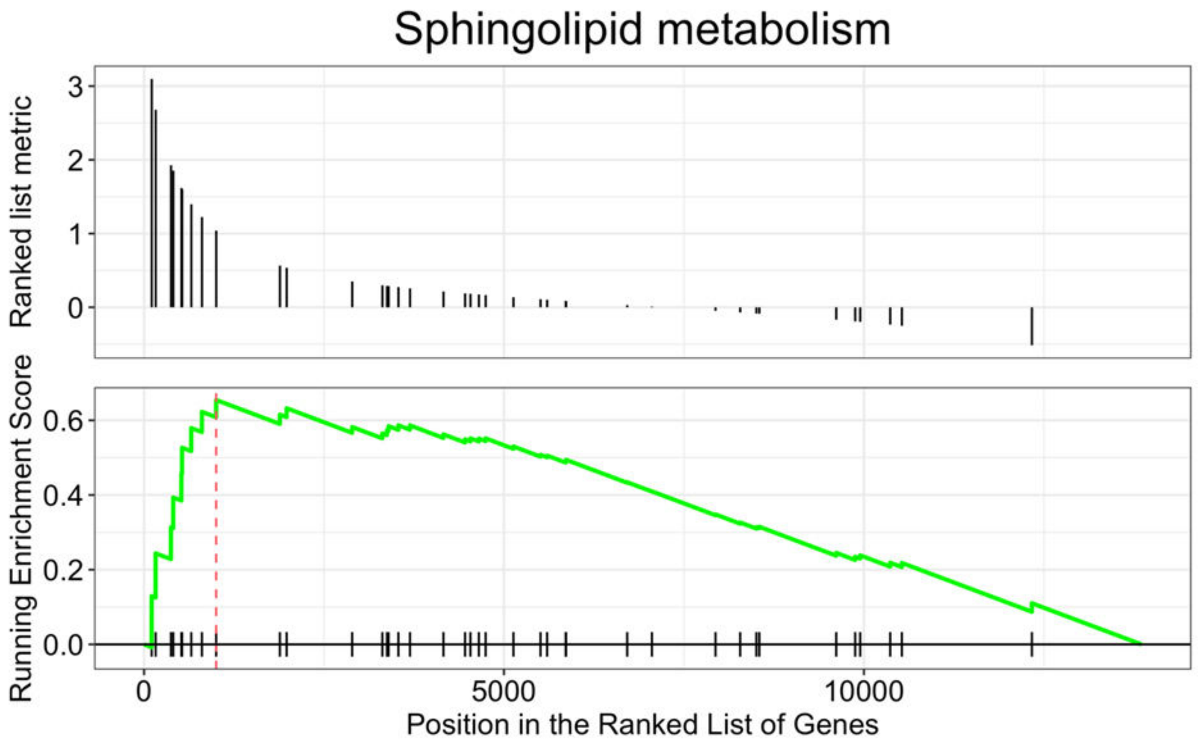

Figure S1 Gene set enrichment analysis (GSEA) on hepatic sinusoidal endothelial cell data sets. Related to Figure 2. (A) Distribution of Log2 fold changes of genes involved in the significantly enriched KEGG pathways predicted by GSEA. Positive Log2 fold changes indicate genes that are upregulated in GF mice relative to the CONV-R group and correspond to activated pathways. (B) GSEA enrichment plot for the sphingolipid metabolism pathway. The upper plot shows the Log2 fold changes (ranked list metric) for the genes included in the sphingolipid metabolism gene set. The lower plot shows the enrichment score as function of the position of genes along the ranked gene set. The maximum value (indicated by the dashed red line) shows the final enrichment score for the pathway.

Figure S2

A

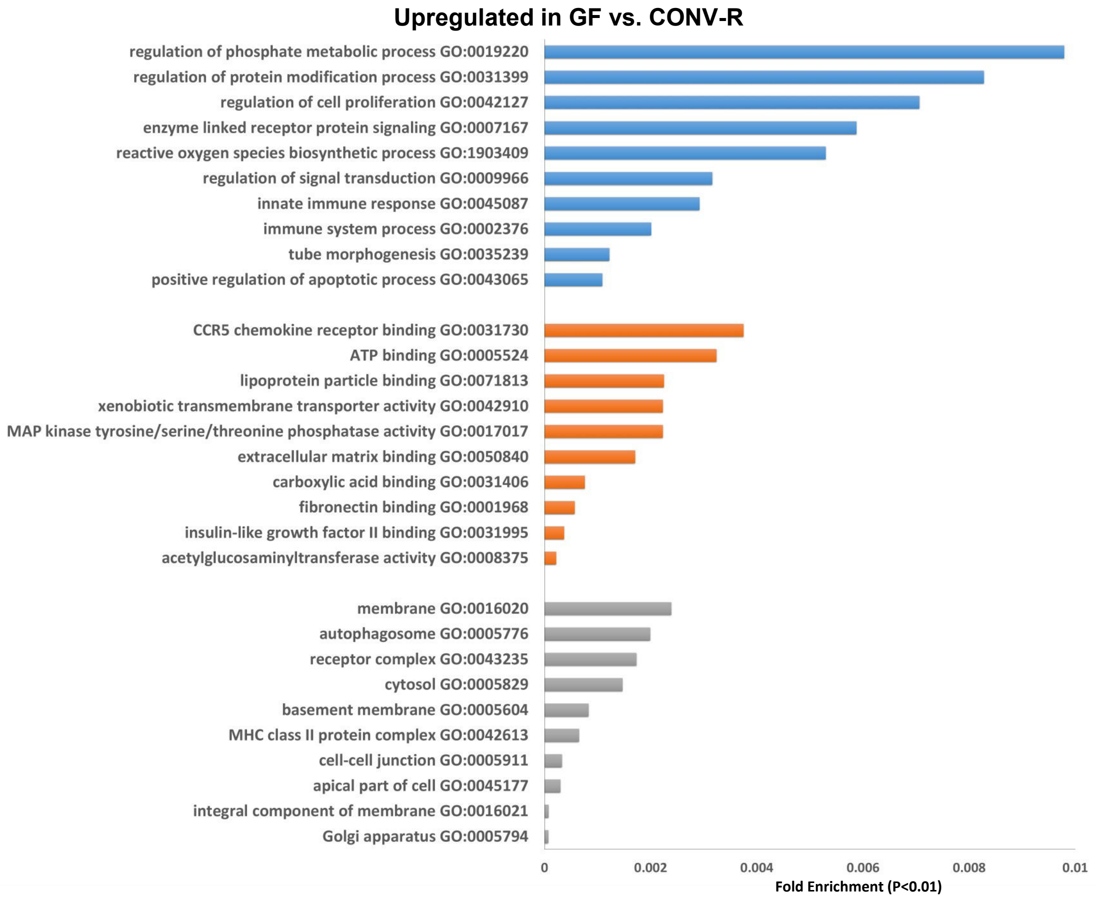

B

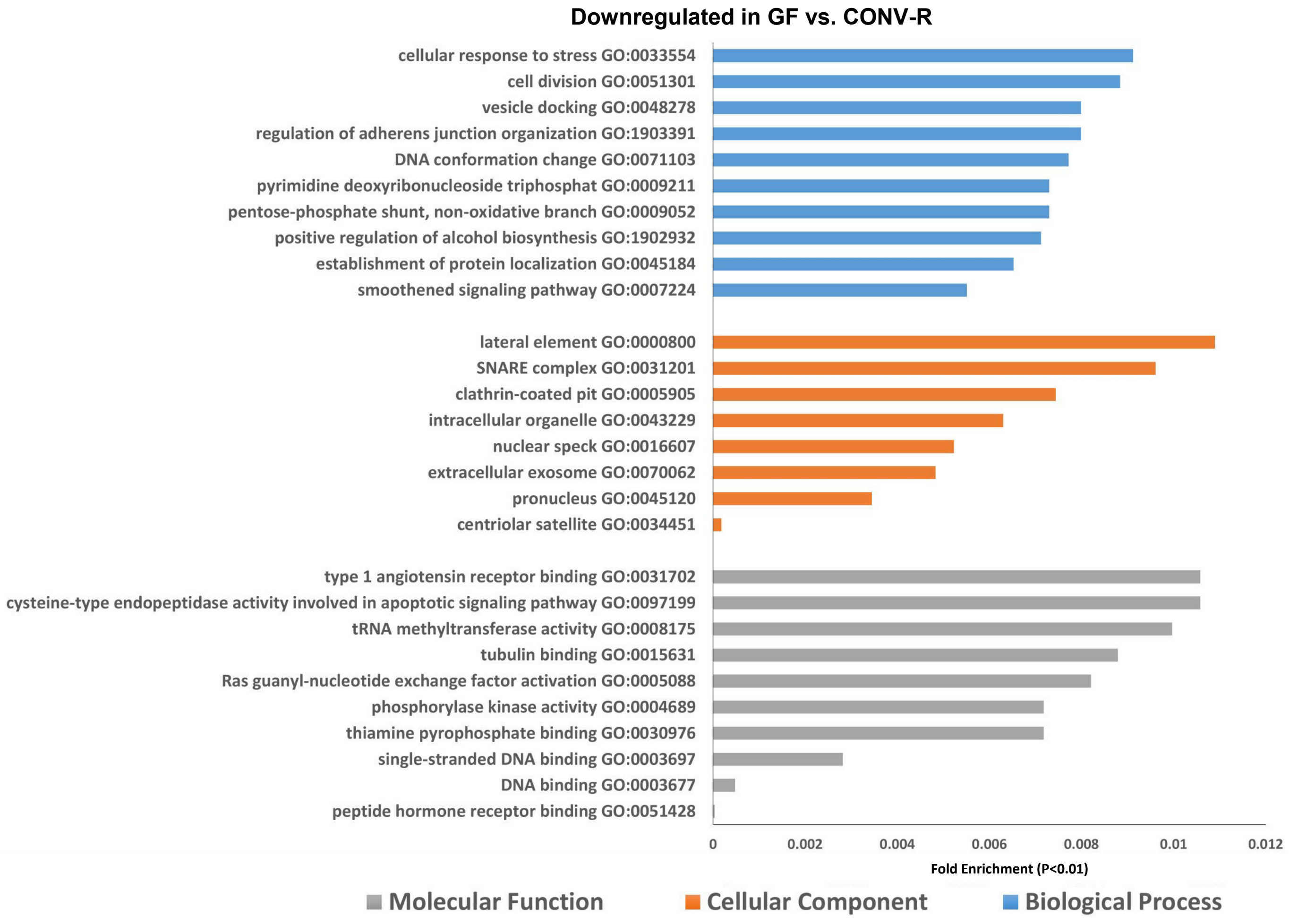

Figure S2 Gene Ontology (GO) term-analysis. Related to Figure 2. Biological process (blue bars), cellular component (orange bars), and molecular function (grey bars). (A) Significantly up-regulated genes in GF vs. CONV-R mice (p.adjust < 0.01). (B) Significantly down-regulated genes in GF vs. CONV-R mice (p.adjust < 0.01). Presentation of top 10 significant GOs per group.

Figure S3

A

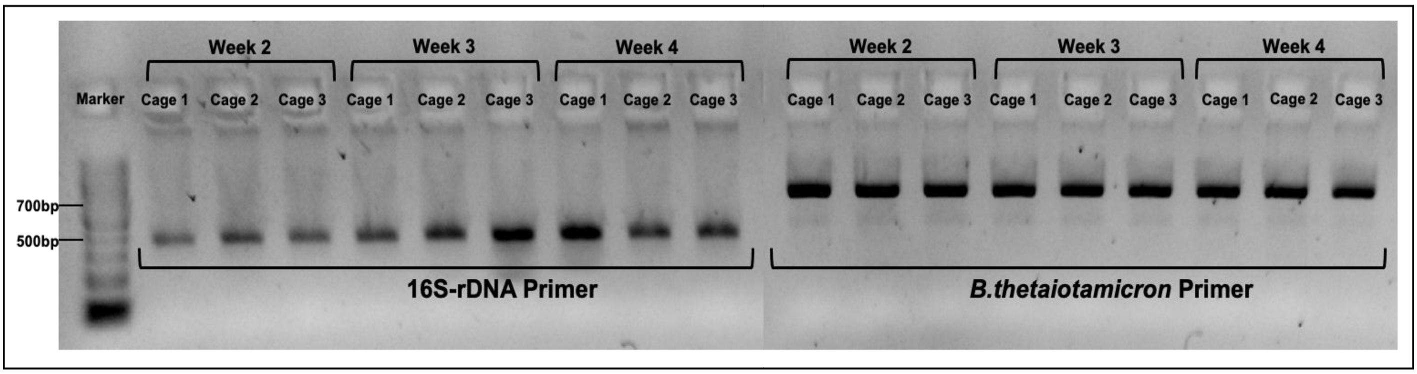

B

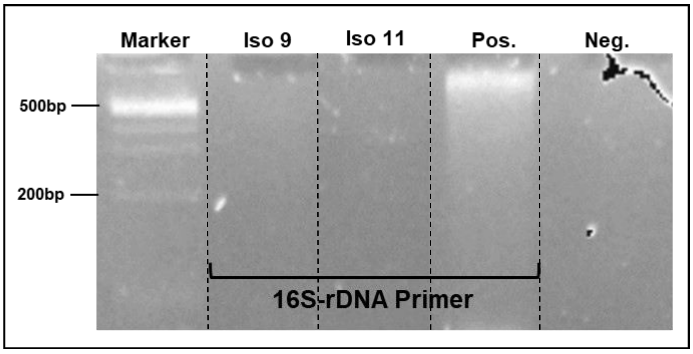

Figure S3 Detection of 16S-rDNA in fecal samples. Related to Figure 3. (A) Control of the monocolonization with *Bacteroides thetaiotaomicron* in 3 cages over four weeks. The left side of the agarose gel shows the 16S-rDNA band while the right side of the agarose gel shows a specific amplicon of the *Bacteroides thetaiotaomicron*. (B) Sterility control on fecal samples collected from the isolators containing C57BL/6J mice of used for colonization with *Bacteroides thetaiotaomicron*.

Figure S4

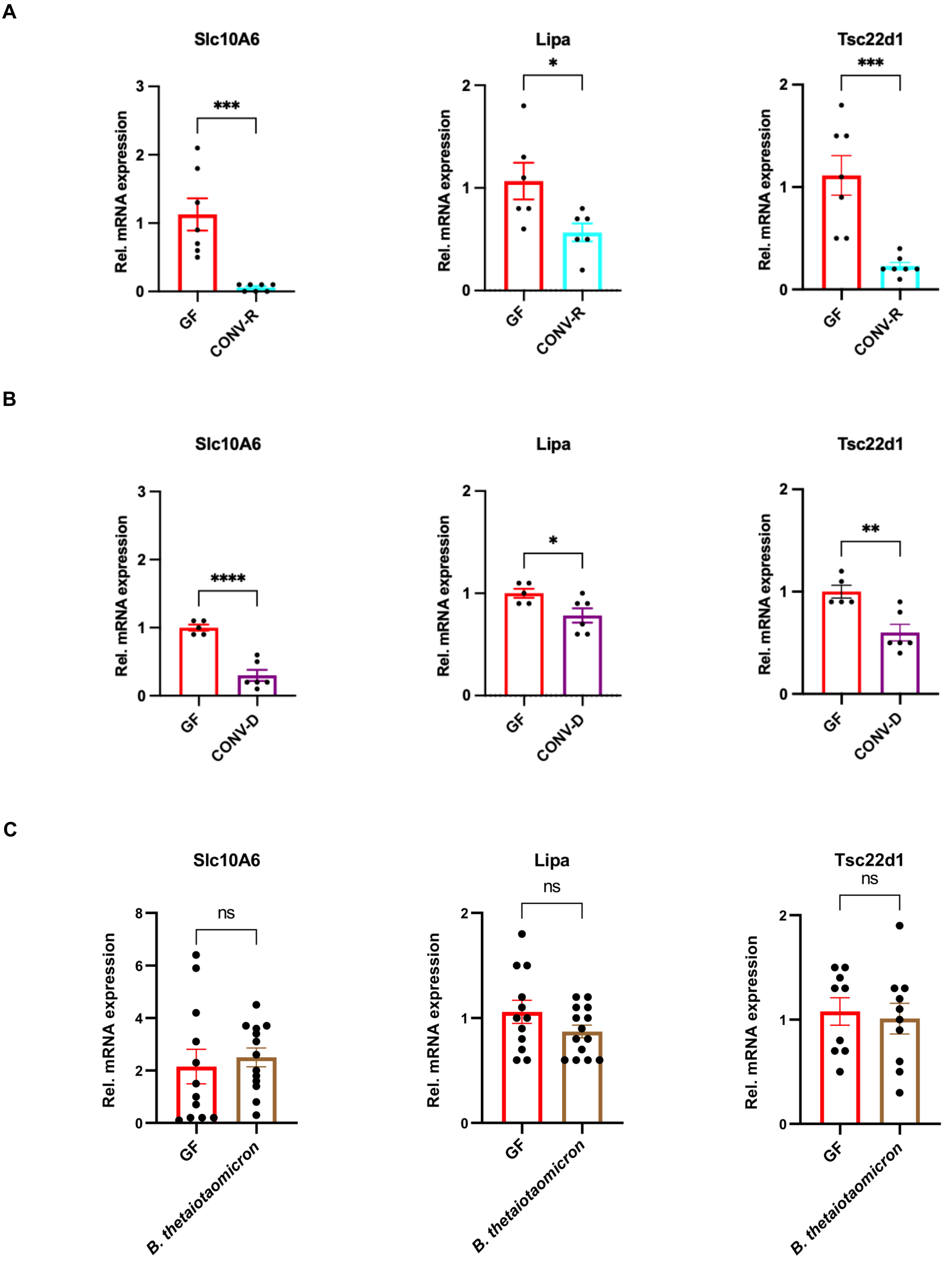

Figure S4 Relative mRNA expression of identified microbiota-regulated genes of the cholesterol flux of MACS- isolated hepatic endothelial cells at various colonization conditions. Related to Figure 2. (A) qRT-PCR quantification of mRNA levels of *SlcA6*, *Lipa*, *Tsc22d1* in MACS-sorted liver endothelial cells of germ-free (GF; red bars; N=6-7 mice per group) vs. conventionally-raised mice (CONV-R, turquoise bars; N=6-7 mice per group); male mice were analyzed. (B) qRT -PCR quantification of mRNA levels of *SlcA6*, *Lipa*, *Tsc22d1* in MACS-sorted liver endothelial cells comparing germ-free (GF; red bars; N=5 mice per group) vs. conventionally derived mice (CONV-D, purple bars; N=6 mice per group); 4 weeks colonization period, mixed groups were analyzed. (C) qRT-PCR quantification of mRNA levels of *SlcA6*, *Lipa*, *Tsc22d1* in MACS-sorted liver endothelial cells comparing germ-free (GF; red bars; N=9-12 mice per group) vs. *Bacteroides thetaiotaomicron*-monocolonized mice (brown bars; N=10-13 mice per group); 4 weeks colonization period, male mice were analyzed. Results are presented as mean +- S.E.M. *=P<0.05; **=P<0.01; ***=P<0.005; ****=P<0.001.

Table S1

**UP-REGULATED IN CONV-R**				
**FUNCTION**	**GENE**	**NAME**	**WITH REGARD TO LIVER AND ENDOTHELIAL CELL PHYSIOLOGY**	**REFERENCES (PMID)**
**Adhesion/Matrix**	**Kank2**	KN Motif And Ankyrin Repeat Domains 2	Connection of talin with microtubule tips KANNK2 is a key molecule linking integrin αVβ5 IACs to MTs	31114072<br>32195252
	**Neurl3**	Neutralized E3 Ubiquitin Protein Ligase 3	unknown	32203131
**Metabolism**	**Alox12**	Arachidonate 12-Lipoxygenase, 12S Type	Gating of lymph endothelial cell barrier<br>Contributing to thrombosis<br>Endothelial cell barrier	24352538<br>27552229<br>29129665
**Cell Adhesion**	**Kazn**	periplakin-binding protein Kazrin	Endothelial junctions	7560895
**UP-REGULATED IN GF**				
**Cell Adhesion**	**Fn1**	Fibronectin 1	Adhesion	28928239
	**Timp4**	Tissue Inhibitor of Metalloproteinases 4	Absence of TIMP-4 ameliorates HFD-induced obesity	28740132
**Cholesterol and Bile acid**	**Slc10A6**	Solute Carrier Family 10 Member 6	Inflammation-dependent up-regulation in mouse liver	26510996<br>24196564
	**Lipa**	Lipase A, Lysosomal Acid Type	Modulation of mTOR pathway; tumor growth and metastasis	25000979<br>28924047
	**Tsc22d1**	TSC22 Domain Family Member 1	TGF-beta regulation of C-type natriuretic peptide expression; Control of cholesterol metabolism	20802130<br>24634828
	**Hdc**	Histidine Decarboxylase	Expressed in liver endothelial cells. Role in cholestatic liver injury	15167966<br>24384130
**Sphingolipid metabolism**	**Abcg2**	ATB Binding Cassette Subfamily G Member 2	Efflux of phototoxins in capillary endothelial cells; highly expressed in liver	16715370<br>23473424<br>20110355
	**S1pr1**	Sphingosine-1-Phosphate Receptor 1	Vascular development; protection against liver ischemia-reperfusion; metastasis and angiogenesis	21281624<br>29273365<br>30377291
	**Acer2**	Alkaline Ceraminidase 2	Endothelial cell proliferation and apoptosis; Promotes growth, invasion and migration of hepatocellular carcinoma cells	10652340<br>32391585
	**Sgms1**	Sphingomyelin Synthase 1	Higher ceramide and lower sphingomyelin in sgms2 knock out mice reduces liver steatosis; regulation of peroxisome proliferator-activated receptor gamma 2<br>Sms1 deficiency decreases sphingomyelin but dramatically increases the levels of glycosphingolipids and reduces atherosclerosis	23640498<br>22580896
**Angiogenesis**	**Map3k6**	Mitogen-Activated Protein Kinase Kinase Kinase 6	Mediates angiogenic and tumorigenic effects via VEGF.	19246638
	**Tle1**	TLE Member 1	Involved in vascular development and angiogenesis; associated with hepatocellular carcinoma	12842817<br>12079511<br>32114961
	**Zbtb16**	Zinc Finger And BTB Domain Containing 16	Proliferation of cultured corneal endothelial cells; inhibition of angiogenesis	17515885<br>20236758
	**Shroom2**	Shroom Family Member 2	Regulation of endothelial sprouting, migration and angiogenesis; regulation	21248203

			of contractility to control endothelial morphogenesis	
	**Glul**	Glutamate-Ammonia Ligase	Supports vessel sprouting and endothelial cell migration	30158707
	**Klf9**	Kruppel Like Factor 9	Regulation of angiogenic properties; control of unfolded protein response; role in hepatic carcinoma	24898819<br>25652467
**Vesicular Transport**	**Syt4**	Synaptotagmin 4	Related family member 5 was associated with Weibel-Palade body exocytosis	30659119
	**Rab3a**	RAS-Associated Protein RAB3A	Control of endothelial tubulogenesis	32569321
**Circadian Rhyhtmicity**	**Klf15**	Kruppel-like Factor 15	Endometriosis and xenobiotic metabolism; toxicity of biliary sight, elimination of steroid hormones; activates hepatitis B replication	32694878<br>24167585<br>21503941<br>17403374
	**Per1**	Period Circadian Regulator 1	Influencing the cell migration and proliferation via the clock gene Dec1	30664860
**Metabolism**	**Qsox1**	Quiescin Sulfhydryl Oxidase 1	Promotes mitochondrial apoptosis of hepatocellular carcinoma cells	32863002
	**Acadsb**	Acyl-CoA Dehydrogenase Short/Branced Chain	Plays a role in gene expression of liver cancer	30176945
	**Xdh**	Xanthine Dehydrogenase	Inactivated Xdh in interferon-gamma-stimulated macrophages, is formed in the liver	7524568<br>17023262
**Signaling**	**Stc1**	Stanniocalcin 1	Influences tumor size in hepatocellular carcinoma; modifies carbohydrate and lipid metabolism	26469082<br>30679516
	**Ndgr1**	N-Myc Downstream Regulated 1	Regulate endothelial cell proliferation and migration	25582201<br>19760510
	**Depp1**	DEPP1 Autophagy Regulator; Decidual Protein Induced By Progesterone	Regulation of hepatic fat metabolism; autophagy activation	29702025<br>24530860
**Inflammation (-)**	**Tsc22d3**	TSC22 Domain Family Member 3, GILZ	Prevents vascular inflammation	24747114
**Membrane Transport**	**Fkbp5**	FKBP Prolyl Isomerase 5	Regulation by corticosteroids	30489628
**Transcriptional Regulation**	**Mbd1**	Methyl-CpG Binding Domain Protein 1	MBD1 complex for the induction of immunotolerance	24464130

Table S1. Top 50 differentially up-regulated genes in the CONV-R vs. GF comparison, sorted by function. Related to Figure 2. Main groups are cell adhesion (blue), cholesterol and bile acid metabolism (yellow), sphingolipid metabolism (red), angiogenesis (orange), and vesicular transport (green).
